# Salivary Cytokine Biomarker Concentrations in Relation to Obesity and Periodontitis

**DOI:** 10.3390/jcm8122152

**Published:** 2019-12-05

**Authors:** Sanna Syrjäläinen, Ulvi Kahraman Gursoy, Mervi Gursoy, Pirkko Pussinen, Milla Pietiäinen, Antti Jula, Veikko Salomaa, Pekka Jousilahti, Eija Könönen

**Affiliations:** 1 Periodontology, Institute of Dentistry, University of Turku, 20520 Turku, Finland; sanna.syrjalainen@utu.fi (S.S.); mervi.gursoy@utu.fi (M.G.); eija.kononen@utu.fi (E.K.); 2 Oral and Maxillofacial Diseases, University of Helsinki, 00014 Helsinki, Finland; pirkko.pussinen@helsinki.fi (P.P.); milla.pietiainen@helsinki.fi (M.P.); 3 Finnish Institute for Health and Welfare, 00271 Helsinki, Finland; antti.jula@thl.fi (A.J.); veikko.salomaa@thl.fi (V.S.); pekka.jousilahti@thl.fi (P.J.); 4 Oral Health Care, Welfare Division, City of Turku, 20101 Turku, Finland

**Keywords:** obesity, periodontitis, cytokine, inflammation, saliva

## Abstract

Systemic low-grade inflammation is associated with obesity. Our aim was to examine the association between obesity and salivary biomarkers of periodontitis. Salivary interleukin (IL)-1-receptor antagonist (IL-1Ra), IL-6, IL-8, IL-10, and tumor necrosis factor (TNF)-α concentrations were measured from 287 non-diabetic obese (body mass index (BMI) of >35 kg/m^2^) individuals and 293 normal-weight (BMI of 18.5–25 kg/m^2^) controls. Periodontal status was defined according to a diagnostic cumulative risk score (CRS) to calculate the risk of having periodontitis (CRS I, low risk; CRS II, medium risk; CRS III, high risk). In the whole population, and especially in smokers, higher IL-8 and lower IL-10 concentrations were detected in the obese group compared to the control group, while in non-smoking participants, the obese and control groups did not differ. IL-1Ra and IL-8 concentrations were higher in those with medium or high risk (CRS II and CRS III, *p* < 0.001) of periodontitis, whereas IL-10 and TNF-α concentrations were lower when compared to those with low risk (CRS I). In multivariate models adjusted for periodontal status, obesity did not associate with any salivary cytokine concentration. In conclusion, salivary cytokine biomarkers are not independently associated with obesity and concentrations are dependent on periodontal status.

## 1. Introduction

Obesity is an increasing health problem in developed countries and is a major risk factor for diabetes, cancer, and cardiovascular diseases [1]. Obesity is linked to both local and systemic inflammation [2,3,4,5]. In obese subjects, there is an elevated release of proinflammatory cytokines into serum derived from either adipocytes, stromal vascular fraction cells, or immune cells in adipose tissue [3,5,6]. This elevated cytokine release in obese individuals is a reversible condition, since even mild weight loss can reduce serum cytokine levels [7].

There is substantial evidence showing a positive association between obesity or weight gain and periodontitis [8,9,10,11]. The underlying mechanisms explaining this association are not completely elucidated, but one proposed mechanism is that the low-grade systemic inflammation related to obesity could expose obese people to infectious diseases [3,4,12]. Pathogenic bacterial biofilms at the gingival margin trigger the initiation of inflammatory processes in periodontal tissues, including the production of chemokines and proinflammatory cytokines [13,14]. Inflammatory cytokines in the periodontium are low-molecular weight proteins secreted from both periodontal tissue and immune cells [13]. They are the main regulators of inflammation and tissue destruction in periodontitis [15], and their levels in saliva predict periodontal disease progression and remission [16]. To our knowledge, the association between salivary cytokine concentrations and obesity and periodontitis has not been examined in humans so far.

In the present study, we used a novel diagnostic method, the cumulative risk score (CRS), to detect periodontal disease based on three biomarkers in saliva [17]. CRS combines *Porphyromonas gingivalis*, interleukin (IL)-1β, and matrix metalloproteinase (MMP)-8, and categorizes individuals with low, medium, or high risk of having periodontitis. Our hypothesis is that low-grade inflammation related to obesity affects the cytokine biomarker concentrations in saliva. In this context, our cross-sectional study aimed to examine the relation between obesity and periodontitis-associated salivary cytokine concentrations in smoking and non-smoking individuals.

## 2. Experimental Section

### 2.1. Study Population

The study consisted of 580 individuals aged 25–74 (mean 55.3) years. They were participants of the Dietary, Lifestyle, and Genetic determinant of Obesity and Metabolic syndrome (DILGOM) study, which was an extension of the population-based National FINRISK2007 health survey to investigate more specifically the effects of diet, lifestyle, and genetic factors on obesity and metabolic syndrome [18,19]. Of the 5024 individuals who participated in the DILGOM study, 287 severely obese (body mass index (BMI) ≥35 kg/m^2^) and 297 normal-weight (BMI 18.5–25 kg/m^2^) controls matched for age and smoking status were included. Exclusion criteria for both cases and controls were diabetes, cardiovascular disease, cancer, or medication for hypercholesterolemia. The protocol of the FINRISK2007 survey included questionnaire data on smoking and other health behaviors, socioeconomic background factors, clinical measurements, and venous blood samples. Participants were categorized by their smoking status as current smokers or non-smokers (smokeless for at least the past 6 months). Height was measured to the nearest 0.1 cm and weight to the nearest 0.1 kg.

### 2.2. Bacterial and Cytokine Measurements from Salivary Samples

Paraffin-stimulated whole saliva samples were collected by expectoration into plastic tubes from the DILGOM study participants. All samples were stored frozen at −70 °C until laboratory analyses. Before analyses, melted samples were gently mixed, and centrifuged at 10,000 × *g* for 5 min. DNA was isolated from the pellet and used in *P. gingivalis* quantification, while the supernatant was used in cytokine determinations.

Salivary concentrations of IL-1β, IL-1 receptor antagonist (IL-1Ra), IL-6, IL-8, IL-10, tumor necrosis factor alpha (TNF–α), and MMP-8 were analyzed with the flow-cytometric Luminex xMAP technique with commercially available kits by Bio-PlexTM 200 (Bio-Rad Laboratories Incorporation, Santa Rosa, CA, USA).

The amounts of *P. gingivalis* were determined with a quantitative real-time PCR (qPCR) assay as previously described [20] with modifications. Reaction mixtures (total volume 20 µL) contained 2 µL of template DNA, 200 nM primers (Thermo Fisher Scientific, Waltham, MA, USA), and 1 × Universal KAPA SYBR FAST qPCR mastermix (KAPA Biosystems, Wilmington, MA, USA) supplemented with 1 × ROX Low reference dye. qPCR analyses were performed with the Mx3005P Real-Time qPCR System (Stratagene, La Jolla, CA, USA) via the following steps: Initial denaturation at 95 °C for 3 min, followed by 40 cycles of 3 s at 95 °C and 20 s at 60 °C. A dissociation curve was generated from one cycle of 1 min at 95 °C, then lowering the temperature gradually to 60 °C, 30 s at 60 °C, then raising the temperature gradually to 95 °C, and 30 s at 95 °C. The data were analyzed with the Mx3005P Real-Time qPCR System software and the results were presented as genomic equivalents (GE)/mL saliva.

For the standard curve, the whole *P. gingivalis waaA* gene, encoding 3-deoxy-Dmanno-oct-2-ulosonic acid (Kdo) transferase, was cloned to pJet1.2/blunt vector (Thermo Fisher Scientific, Waltham, MA, USA). The cloned fragment was PCR-amplified in the reaction containing 500 nM primers (Fwd-ATGCGATTCCTTTTCAG and Rew-CTATTTCATGATTCGGTG), 200 µM dNTPs, Phusion DNA Polymerase (Thermo Fisher Scientific, Waltham, MA, USA) 0.04 U/µL, 1 × Phusion High-Fidelity Buffer, and 10 ng of chromosomal DNA of *P. gingivalis* strain W50. The cycling conditions followed the manufacturer’s instructions. The purified PCR fragment was ligated into pJet1.2/blunt vector with CloneJET PCR Cloning Kit (Thermo Fisher Scientific, Waltham, MA, USA) according to the manufacturer’s instructions and ligation mixture was transformed into *Escherichia coli* DH5a competent cells. The correct insert was verified by sequencing. The constructed plasmid, pJet1.2/blunt-Pg, was linearized with FastDigest HindIII restriction enzyme (Thermo Fisher Scientific, Waltham, MA, USA) and used for a tenfold dilution series for the qPCR analysis. The plasmid copy number was determined with “DNA Copy Number and Dilution Calculator” (www.thermofisher.com).

### 2.3. Periodontal Status Assessment Based on Cumulative Risk Score (CRS)

Periodontal status of the study population (*n* = 580) was defined according to CRS as described in detail by Gursoy et al. [17]. Briefly, for calculating CRS, the salivary concentrations of *P. gingivalis*, IL-1β, and MMP-8 were independently divided into tertiles and each participant was categorized to one of the three tertiles according to the level of the biomarker in the saliva. The person’s cumulative score was calculated by multiplication of three biomarkers’ tertile values. Based on these calculations, the study participants were categorized into three periodontal status groups, as follows: CRS I: low risk; CRS II: medium risk; CRS III: High risk [17,21,22].

### 2.4. Statistical Analyses

All statistical analyses were conducted using the IBM SPSS Statistic 23.0 software (IBM, Armonk, North Castle, NY, USA). In descriptive statistics, continuous variables were reported as means and standard deviations and the differences between the groups were analyzed by independent *t*-test. Categorical variables were reported as the number of individuals and as percentages. Differences between the groups in categorical variables were analyzed using the chi-square test. Statistical significance was set at a *p*-value of < 0.05.

Results of two saliva samples were not included in analyses due to the low sample quality. Due to skewed distribution, salivary cytokine concentrations were reported as medians and interquartile ranges. Differences of salivary cytokine concentrations between the obese and normal-weight participants and a pairwise comparison between the groups among different CRS categories were conducted using the Mann–Whitney *U* test. A *p*-value significance level of < 0.05 was used. Multinomial logistic regression was used to determine whether salivary cytokines were associated with obesity, before and after adjusting the model for smoking and periodontal status (CRS). For multinomial logistic regression, cytokine concentrations were converted into tertiles, and the lowest tertile was used as the reference group.

### 2.5. Ethical Issues

The study was conducted in accordance with the Declaration of Helsinki and approved by the Ethical Committee of the Hospital District of Helsinki and Uusimaa. Written informed consent was obtained from each participant.

## 3. Results

There was no difference in age, gender, or smoking status between the two study groups, whereas mean BMI was significantly higher in the obese (39.0 kg/m^2^) than in the normal-weight (22.9 kg/m^2^) groups (*p* < 0.001) (Table 1). Obese individuals were lower educated (*p* = 0.005) and were more likely to have periodontal disease than their controls (*p* = 0.015).

In the whole population and in smokers, the salivary concentrations of IL-8 were higher (whole population *p* = 0.033; smokers *p* = 0.005) and those of IL-10 were lower (whole population *p* = 0.022; smokers *p* = 0.018) in the obese group than in their controls (Table 2). Other salivary cytokine concentrations did not differ according to weight status. In non-smokers, there was no difference in salivary cytokine concentrations between the groups.

When the study participants were stratified only according to their periodontal status and not by their weight, IL-1Ra and IL-8 concentrations were higher in those with medium or high risk (CRS II and CRS III, *p* < 0.001) of periodontitis, whereas IL-10 and TNF-α concentrations were lower when compared to those with the low risk (CRS I). After weight was taken into account, obese individuals with CRS I had lower IL-10 (*p* = 0.043) and TNF-α (*p* = 0.35) concentrations than their normal-weight controls, while obese individuals with CRS III had higher IL-6 concentrations (*p* = 0.011). Other cytokine concentrations in saliva did not differ between the obese and control groups according to their periodontal status (Table 3).

The multinomial regression model revealed a significant association between obesity and IL-10 concentrations, but the significance was lost after the model was adjusted for smoking and periodontal status (Table 4).

## 4. Discussion

The main finding of the present study was that, despite periodontal status being worse in obese individuals (BMI of ≥35kg/m^2^) compared to normal-weight controls (BMI of 18.5–25 kg/m^2^), the association was not consistently reflected in salivary cytokine concentrations. Instead, the concentrations were merely affected by periodontal status rather than obesity. To our knowledge, this was the first study to investigate potential associations of salivary cytokines with obesity by taking periodontal and smoking states into account.

The relatively large sample size, including 287 severely obese and 293 normal-weight individuals, allowed us to make reliable comparisons of cytokine concentrations in saliva between these groups. However, the cross-sectional study design does not provide any information about the causality between periodontitis, obesity, and cytokines. In addition, the relatively large age range of the study population may have displayed an underestimated effect, since the immune response undergoes remodeling with age. To define the presence of periodontal disease, a novel salivary diagnostic tool, CRS, was used [17]. Its capability has been validated in independent populations twice, showing that the CRS index is more strongly associated with moderate to severe periodontitis than any of the salivary biomarkers alone [21,22]. Finally, as part of the sample collection protocol of this survey study, stimulated saliva samples were collected. The protein concentration was higher in the unstimulated saliva samples than the stimulated saliva samples, however, the unstimulated saliva samples possessed greater inter- and intraindividual variation than the stimulated saliva samples [23].

According to the present study, obese individuals expressed enhanced IL-8 and IL-1Ra but reduced IL-10 concentrations in saliva when compared to normal-weight individuals. This finding was observed especially in smokers. Studies dealing with salivary cytokines in relation to weight are sparse. In a recent small-scale study (*n* = 44), TNF-α concentrations in saliva were shown to be significantly higher in obese than non-obese adults [24]. In obese and non-obese children, on the other hand, salivary TNF-α concentrations did not differ [25]. As both obesity and periodontitis are inflammatory states, it was proposed that obesity-related inflammation could predispose obese subjects to periodontal tissue destruction [12,26]. In the present study, obese individuals displayed mainly CRS II and III, indicating worsened periodontal conditions, more frequently than their normal-weight controls. Nevertheless, there were no consistent differences in the salivary cytokine concentrations between the groups.

In periodontitis, cytokines are mainly released from periodontal tissues after pathogenic bacterial recognition [13,14]. There is also some evidence that adipose tissue-derived cytokines act in a paracrine more than an endocrine manner, and hence do not contribute to cytokine concentrations in the oral cavity [27,28]. It is therefore possible that the local infection of the periodontium has such a strong effect on salivary cytokine concentrations that it overpowers the systemic influence of obesity in statistical analyses. Still, obesity may reinforce the inflammatory response to periodontal pathogens in the periodontium, resulting in an increased susceptibility to periodontitis. It is also possible that the link between obesity and periodontitis is not explained by inflammatory factors.

Obesity and periodontitis share other risk factors, including low socioeconomic status [29]. In the present study, obese individuals were less educated than their controls. This is in line with studies linking educational status to health behaviors as well as eating habits to the prevalence of periodontal disease [29,30,31]. Obesity-related comorbidities, for example, diabetes mellitus, which is a well-known risk factor for periodontitis [26], could also explain the association between obesity and periodontitis. In our study, diabetic patients were excluded, but the obese individuals may still have insulin resistance, a prediabetic state, which is also a proposed risk factor for periodontitis [32].

In addition to obesity, an unhealthy diet causing weight gain plays a role in the obesity-related inflammatory burden [33,34,35]. In an experimental rodent model, it was shown that obese rats fed a high-fat and high-carbohydrate diet mimicking the Western diet (known as the “cafeteria diet”) caused significantly more advanced alveolar bone loss when compared to non-obese counterparts [34]. In another rodent model, it was observed that a diet enriched with saturated fat was associated with higher inflammatory potential and tissue destruction when compared to a diet high in unsaturated fat in obese mice [34]. Therefore, in future studies regarding obesity and periodontitis in humans, it would be of interest to include the dietary composition of obesity in the analyses.

## 5. Conclusions

In conclusion, although obese individuals may be prone to have periodontal disease, obesity does not lead to altered cytokine concentrations in saliva. The associations between obesity and salivary cytokine concentrations may be explained by periodontal status and smoking.

## Figures and Tables

**Table 1:** Characteristics of the study groups according to their weight status. Significant differences (*p* < 0.05) are presented in bold.

Demographic and Clinical Parameters	Obese *n* = 287	Normal Weight *n* = 293	*p*
Age, mean (SD)	55.0 (11.9)	55.5 (12.0)	0.604
Males, *n* (%)	98 (34.1)	98 (33.4)	0.859
Smokers, *n* (%)	51 (17.8)	47 (16.0)	0.578
Education level, *n* (%)			
Low	105 (37.1)	74 (25.3)	**0.005**
Average	94 (33.2)	104 (31.7)	
High	84 (29.7)	115 (39.2)	
BMI, mean (SD)	39.0 (4.1)	22.9 (1.6)	**<0.001**
Periodontitis, *n* (%)			
Cumulative Risk Score (CRS) I	58 (20.2)	80 (27.3)	**0.015**
Cumulative Risk Score (CRS) II	82 (28.6)	97 (33.1)	
Cumulative Risk Score (CRS) III	147 (51.2)	116 (39.6)	

*p*-values: Independent samples *T*-test (age, body mass index (BMI)) and chi-square test (gender, smoking status, educational level, periodontitis).

**Table 2:** Salivary cytokine concentrations (pg/mL) in the study population and as divided according to smoking status. Significant differences (*p* < 0.05) are presented in bold.

	Whole Population			Smokers			Non-Smokers		
	Obese *n* = 285	Normal Weight *n* = 293	*p*	Obese *n* = 50	Normal Weight *n* = 47	*p*	Obese *n* = 235	Normal Weight *n* = 246	*p*
IL-1Ra	7979 (8712)	7782 (8962)	0.097	7018 (7885)	4877 (4915)	0.036	8566 (9141)	8098 (9136)	0.307
IL-6	3.6 (5.2)	3.3 (4.2)	0.095	3.6 (5.7)	3.6 (6.1)	0.948	3.6 (5.2)	3.2 (4.0)	0.071
IL-8	378 (472)	300 (365)	**0.033**	345 (431)	241 (152)	**0.005**	380(472)	375 (400)	0.257
IL-10	1.5 (3.0)	2.0 (3.7)	**0.022**	1.7 (3.2)	2.7 (6.5)	**0.018**	1.5 (2.9)	1.9 (3.7)	0.132
TNF-α	10.4 (17.4)	12.6 (18.8)	0.103	10.7 (18.5)	18.3 (23.4)	0.094	10.1 (17.5)	12.4 (17.7)	0.291

All values are given as medians (interquartile ranges). *p*-values: Mann–Whitney *U* test.

**Table 3:** Salivary cytokine concentrations (pg/mL) in the study population divided according to periodontal status. Significant differences (*p* < 0.05) are presented in bold.

	CRS I			CRS II			CRS III		
	Obese *n* = 58	Normal Weight *n* = 80	*p*	Obese *n* = 82	Normal Weight *n* = 97	*p*	Obese *n* = 147	Normal Weight *n* = 116	*p*
IL-1Ra	4419 (2829)	3727 (3497)	0.064	6582 (5478)^a.^	7224 (5402)^a^	0.666	12136 (9387)^a.b^	13045 (10240)^a,b^	0.212
IL-6	3.6 (4.3)	4.5 (4.7)	0.341	2.9 (3.3)	3.0 (3.0)^a^	0.993	4.8 (6.8)^a,b^	3.0 (5.5)	**0.011**
IL-8	169 (127)	168 (125)	0.440	274 (292)^a^	284 (242)^a^	0.682	573 (443)^a,b^	566 (464)^a,b^	0.784
IL-10	3.5 (5.8)	5.7 (8.1)	**0.043**	1.5 (2.8)^a^	2.0 (3.0)^a^	0.078	1.3 (2.2)^a^	1.2 (1.9)^a,b^	0.698
TNF-α	14.4 (23.9)	26.4 (26.4)	**0.035**	9.8 (14.5)^a^	12.4 (13.9)^a^	0.460	8.9 (15.8)^a,b^	10.3 (10.9)^a^	0.841

All values are given as medians (interquartile ranges). In each CRS group, cytokine concentrations were compared between obese and normal-weight groups (*p*-values). Superscript letters indicate significant differences between the CRS groups in obese and normal-weight individuals as follows: a) A difference in CRS I and b) a difference in CRS II. *P*-values and superscripts: Mann–Whitney *U* test.

**Table 4:** Associations of salivary IL-1Ra, IL-6, IL-8, IL-10, and TNF-α tertiles with obesity, before and after adjusting the model for smoking and periodontal status (CRS). Significant associations (*p* < 0.05) are presented in bold.

	Middle Tertile		Highest Tertile	
	Unadjusted	Adjusted	Unadjusted	Adjusted
IL-1Ra	1.5 (1.0–2.3), **0.033**	1.3 (0.9–2.1), 0.194	1.5 (0.9–2.2), 0.06	1.1 (0.7–1.8), 0.722
IL-6	1.3 (0.9–1.9), 0.222	1.3 (0.8–1.9), 0.247	1.2 (0.8–1.8), 0.334	1.2 (0.8–1.8), 0.359
IL-8	1.1 (0.7–1.6), 0.760	0.9 (0.5–1.3), 0.514	1.4 (0.9–2.1), 0.114	0.9 (0.6–1.6), 0.852
IL-10	0.8 (0.5–1.2), 0.264	0.8 (0.5–1.2), 0.287	0.7 (0.4–0.9), **0.042**	0.8 (0.5–1.2), 0.247
TNF-α	0.8 (0.5–1.2), 0.263	0.8 (0.5–1.2), 0.306	0.7 (0.5–1.1), 0.114	0.8 (0.5–1.2), 0.327

Odds ratios (95% confidence intervals) and *p*-values: Multinomial logistic regression model.
